# Palaeoecological inferences for the fossil Australian snakes *Yurlunggur* and *Wonambi* (Serpentes, Madtsoiidae)

**DOI:** 10.1098/rsos.172012

**Published:** 2018-03-14

**Authors:** Alessandro Palci, Mark N. Hutchinson, Michael W. Caldwell, John D. Scanlon, Michael S. Y. Lee

**Affiliations:** 1 South Australian Museum, Adelaide, South Australia, Australia; 2 College of Science and Engineering, Flinders University, Adelaide, South Australia, Australia; 3 School of Biological Sciences, University of Adelaide, Adelaide, South Australia, Australia; 4 Department of Biological Sciences, University of Alberta, Edmonton, Alberta, Canada; 5 School of Biological, Earth and Environmental Sciences, University of New South Wales, Kensington, New South Wales, Australia

**Keywords:** madtsoiid snakes, ecology, labyrinth, geometric morphometrics, principal components analysis, canonical variates analysis

## Abstract

Madtsoiids are among the most basal snakes, with a fossil record dating back to the Upper Cretaceous (Cenomanian). Most representatives went extinct by the end of the Eocene, but some survived in Australia until the Late Cenozoic. *Yurlunggur* and *Wonambi* are two of these late forms, and also the best-known madtsoiids to date. A better understanding of the anatomy and palaeoecology of these taxa may shed light on the evolution and extinction of this poorly known group of snakes and on early snake evolution in general. A digital endocast of the inner ear of *Yurlunggur* was compared to those of 81 species of snakes and lizards with known ecological preferences using three-dimensional geometric morphometrics. The inner ear of *Yurlunggur* most closely resembles both that of certain semiaquatic snakes and that of some semifossorial snakes. Other cranial and postcranial features of this snake support the semifossorial interpretation. While the digital endocast of the inner ear of *Wonambi* is too incomplete to be included in a geometric morphometrics study, its preserved morphology is very different from that of *Yurlunggur* and suggests a more generalist ecology. Osteology, palaeoclimatic data and the palaeobiogeographic distribution of these two snakes are all consistent with these inferred ecological differences.

## 1. Introduction

Several recent studies have shown a close correlation between the shape of part or all of the inner ear apparatus (sacculus, lagena and semicircular canals) and ecological preferences in modern squamate reptiles, i.e. lizards and snakes (e.g. [1–3]). In addition, these studies [1–3] were focused on testing whether it is possible to draw inferences about the palaeoecology of extinct taxa by comparing the morphology of their inner ears with that of modern species with recognized ecological preferences. It was demonstrated that ecology has heavily influenced the morphology of the inner ear throughout snake evolution [1], though phylogeny also plays a part [3]. A study of the semicircular canals among Greater Antillean *Anolis* lizard species [2] found ‘ecomorph’ as the most important covariate of morphology; fossil taxa were found to have different canal shapes and inferred to possess different ecological preferences from modern species. The use of three-dimensional geometric morphometrics to quantify inner ear shape variables and investigate correlations to ecological preferences in both modern and fossil taxa of squamate reptiles is thus showing promising results, particularly when balanced against phylogenetic hypotheses [2].

The Madtsoiidae is a totally extinct snake lineage, which lived between the Upper Cretaceous (Cenomanian) and the Late Pleistocene. This family mostly had a Gondwanan distribution [4–10], and by the end of the Eocene disappeared everywhere except in Australia and Argentina [11,12]. These snakes were initially considered to be closely related to pythons and boas (e.g. [13,14]), but most recent phylogenetic analyses placed them in a more basal position, which makes them a potentially pivotal group for our understanding of snake origins and evolution [7,8,15–18] (but see also [19–21], who place madtsoiids within Alethinophidia).

**Figure 1.**
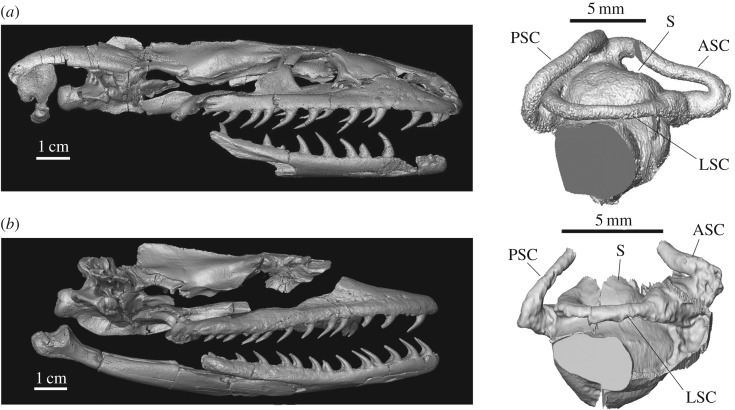
Digital renderings of the reconstructed skulls (based on known elements) and of the inner ear endocasts of (*a*) *Yurlunggur* sp. (QMF45391, QMF45111) and (*b*) *Wonambi naracoortensis* (SAMA P30178A, SAM P27777). Note that the skulls are composites of at least two individuals, and some of the bones were scaled to match the rest (for example, the braincase of *Wonambi* was scaled up 17%). The inner ear endocast of *Yurlunggur* was mirrored for ease of comparison. Abbreviations: ASC, anterior semicircular canal; LSC, lateral semicircular canal; PSC, posterior semicircular canal; S, saccular region.

*Wonambi naracoortensis*, from Pliocene and Pleistocene deposits in southern Australia [7,22–24], and *Yurlunggur* spp. [8], from the Late Oligocene to Middle Miocene deposits of Riversleigh, in northern Queensland, are the two best known of all madtsoiid snakes, with most cranial and postcranial elements known and described. Owing to their completeness, they represent the best sources of information on the anatomy of this extinct lineage, and may help shed light on the ecology and habitat preferences of this group. Therefore, we micro-computed tomography (CT) scanned the braincases of both snakes and obtained digital endocasts of their inner ears (figure 1). The most complete endocast, that of *Yurlunggur* sp. (QMF 45 111–45 391; see end of Material and methods section for the list of institutional abbreviations) was then compared with those of 81 extant species of squamate reptiles of known ecological preference using three-dimensional geometric morphometrics. The less complete inner ear of *Wonambi* (SAMA P30178A) could not be landmarked, and thus was compared only based on gross morphology to the inner ears of *Yurlunggur* and other squamates. Other cranial and postcranial features were also examined to further test our conclusions regarding the palaeoecology of these taxa.

Because the effect of ontogenetic variation on the shape of inner ear endocasts of squamate reptiles is currently unknown, we also provide here, for the first time, a quantitative analysis of ontogenetic trajectories in a selection of eleven taxa, inclusive of both lizards and snakes. The results could be of use to researchers with inner ear data for immature specimens, either extinct or extant.

## 2. Material and methods

Landmark coordinates for the inner ears of 79 squamate reptiles were taken from the electronic supplementary material in [3,25] (i.e. all except *Teretrurus* and *Dinilysia*; *Teretrurus* was replaced in this study by another uropeltid snake, *Rhinophis*, which we consider a more derived exemplar for this group; and *Dinilysia* was excluded because of its unknown ecology). The inner ear of *Platecarpus* was taken from Yi & Norell [1,26]. Micro-CT scan data for *Atractaspis*, *Rhinophis, Wonambi* and *Yurlunggur* (not sampled in [3]) were acquired using a Skyscan 1076 at Adelaide Microscopy (University of Adelaide, Adelaide, South Australia) (electronic supplementary material S1, table S1; see this table also for a list of specimen numbers and taxonomic authorities). The software NRecon (Bruker microCT) was used to reconstruct stacks of images (.bmp) from the micro-CT scan data, and a digital endocast of the right inner ear was produced for each of these specimens via segmentation in Avizo v. 9.0 (Thermo Scientific™).

Three of the four new digital endocasts (the inner ear of *Wonambi* was not landmarked due to incompleteness) were landmarked in Landmark Editor v. 3.6 [27], following the procedure outlined in [3] (see electronic supplementary material, S2).

A recent study on mammalian bony labyrinths [28] pointed out that digital thresholding of CT scan data, the procedure used to obtain surface renderings of anatomical structures to be analysed using geometric morphometrics, can lead to artificial variation in the thickness of the semicircular canals. For this reason, [28] recommended to digitize landmarks on a centreline that runs along the canals rather than on their surface. However, this happens only when considerably different thresholds are used in the different specimens [28], and because in our case all surface files but one (*Platecarpus*, see above) were extracted by the same person, such large inconsistencies in the thresholding can be excluded. Moreover, because our landmarking scheme makes use of points on the sacculus and ampulla and not only on the canals, reducing the inner ear endocast to its midline skeleton via thinning of the volume [28] was not a valid option.

Measurement error in the placing of our selection of landmarks on the inner ear endocasts was tested and confirmed to be negligible in the previous study that used the same core dataset and landmark scheme [3], and will not be further discussed here.

**Figure 2.**
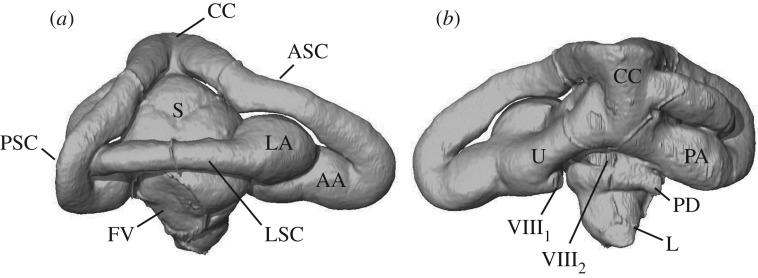
Main anatomical regions of the inner ear endocast of a squamate reptile (the colubroid snake *Psammodynastes pulverulentus*), in (*a*) lateral and (*b*) medial view. Abbreviations: AA, anterior ampulla; ASC, anterior semicircular canal; CC, common crus; FV, fenestra vestibuli (=fenestra ovalis); L, lagenar region; LA, lateral ampulla; LSC, lateral (=horizontal) semicircular canal; PA, posterior ampulla; PD, perilymphatic duct (part); PSC, posterior semicircular canal; S, saccular region; U, utriculus; VIII_1_, anterior branch of the auditory nerve (part); VIII_2_, posterior branch of the auditory nerve (part).

All anatomical terms adopted are from [29,30], and illustrated in figure 2. The landmark configurations of all specimens are provided in electronic supplementary material, S3 (‘tps’ format). The landmark configurations (eight fixed landmarks, 40 sliding semilandmarks) were scaled and aligned with a Procrustes superimposition using the R v. 3.3.2 [31] package geomorph v. 3.0.3 [32]. Analyses of the dataset were carried out in R v. 3.3.2 [31] using the packages geomorph v. 3.0.3 [32], Morpho v. 2.4.1.1 [33], phytools [34] and phylotools v. 0.1.2 [35], and in MorphoJ v. 1.06d [36].

A canonical variates analysis (CVA) was used to display the separation of the various groups in shape space. This analysis was first run in R using the package Morpho v. 2.4.1.1 [33] with jackknife cross-validation (1000 replicates), and then plots and diagrams were produced in MorphoJ [36].

We tested for the presence of a consistent ontogenetic pattern in the growth of the inner ears of eleven juvenile–adult pairs of selected squamates (inclusive of both lizards and snakes: *Acrochordus arafurae*, *Anilios* (*Ramphotyphlops*) *bicolor*, *Aspidites ramsayi*, *Boiga irregularis*, *Candoia carinata*, *Cerberus rhynchops*, *Ctenophorus decresii*, *Ctenotus spaldingi*, *Cylindrophis ruffus*, *Notechis scutatus*, *Varanus gilleni*). The pairs of inner ear endocasts were landmarked using the same scheme adopted for the other specimens and described in electronic supplementary material, S3. We then ran a principal components analysis (PCA) using this morphometric data, and the ontogenetic trajectories between juveniles and adults of each pair were examined in the morphospace defined by the first three principal components (PCs).

The phylogenetic tree adopted for the various phylogenetic tests (phylogenetic signal, phylogenetic ANOVA and phylogenetic PCA) using extant taxa was obtained from Zheng & Wiens [37], with unsampled species pruned using Mesquite v. 3.2 [38], but all branch lengths were retained. Whenever one of our selected species was missing in the tree, we selected a close relative (see electronic supplementary material, S2). Three additional trees inclusive of the fossil taxa *Platecarpus* and *Yurlunggur* (three alternative positions: see below) were obtained after insertion of these fossils into the tree of extant species using the editing tools of Mesquite v. 3.2 [38]. *Platecarpus* was positioned according to the topology in [39] and inserted midway along the relevant branch, i.e. halfway between the node representing the most recent common ancestor of extant snakes (Ophidia) and that of the clade ((Anguimorpha, Iguania), Ophidia). In a similar way, *Yurlunggur* was inserted in three different positions, resulting in three alternative tree topologies to accommodate phylogenetic uncertainty: (1) *Yurlunggur* was placed as a stem ophidian (Tree 1) (e.g. [18]); (2) *Yurlunggur* was placed as a stem alethinophidian (Tree 2) (e.g. [40]); (3) *Yurlunggur* was placed within Alethinophidia (Tree 3) (e.g. [21]), in particular, in a position just above *Anilius* and *Tropidophis* (see electronic supplementary material S2, figure S2). *Platecarpus tympaniticus* was assigned a tip age of 81 Myr [41], while *Yurlunggur* sp. was assigned a tip age of 23 Myr [8,42].

We assessed the effect of phylogenetic signal using the function ‘physignal’ in the package geomorph v. 3.0.3 [32], using a phylogeny with branch lengths and divergence times between the 80 sampled extant taxa from Zheng & Wiens [37] (unsampled terminal taxa were pruned). The fossil *Platecarpus* was inserted into this phylogeny based on [39], and *Yurlunggur* was inserted in three alternative positions based on [18,21,40] (see above). Thus, three supertrees were used to take into account the uncertainty regarding the placement of *Yurlunggur.* Phylogenetic signal was tested using each of the three alternative trees inclusive of all 82 taxa. The test was performed with 10 000 random permutations.

We carried out non-phylogenetic and phylogenetic Procrustes analyses of variance (ANOVA) using a randomized residual permutation procedure (10 000 iterations) [43–45] to test for correlation between shape and groups defined based on ecological habits. The phylogenetic ANOVA was run only using the tree of 80 extant species, because that is the tree where ecological data are available for all taxa and where there is less uncertainty about phylogenetic relationships.

We first used an ordinary (i.e. non-phylogenetic) PCA to see where *Yurlunggur* is located in shape space compared to other taxa based only on morphology. We then ran a phylogenetically informed PCA (phylogenetic PCA or PPCA) to provide a correction for the distribution in the shape space of the taxa that may be affected by phylogenetic signal. The phylogenetic PCA was carried out in the R package phytools v. 0.6–00 (function phyl.pca) [34,46] and the model of evolution was set to uniform Brownian motion.

We tested for a possible correlation between centroid size (CS, an index of overall size) [47] and first principal components (PC1) from both ordinary and phylogenetic PCAs using Pearson, Kendall and Spearman methods [48]. PC1 was selected because in tests of multivariate allometry PC1 is the most appropriate PC as it treats all variables equally [49], and because in biological datasets size is typically the dominant factor contributing to variation, and PC1 is that direction of multidimensional space that accounts for the greatest proportion of variance [49].

We included a classification (group affinity) test using the ‘typprobClass’ function in the package Morpho v. 2.4.1.1 [33], which calculates the typicality probability that a given species belongs to any given group (in this case ecological categories) based on the Mahalanobis distance [50]. This was meant to ascertain which ecological group *Yurlunggur* is closest to, based on the scores of the first two PCs (tests performed on scores from both ordinary and phylogenetic PCAs; ecological groups were defined for all taxa except *Yurlunggur*).

Information about the ecological preferences of the selected species (except *Yurlunggur*, which was left as unknown) was obtained from a survey of the literature (electronic supplementary material S4, table S2). We adopted the same five ecological categories of [3], keeping in mind the same caveats: (i) *generalist*, squamates that are commonly found in a variety of habitats and typically forage on the ground surface; (ii) *arboreal*, species that spend most of their time basking and foraging in trees or shrubs; (iii) *fossorial*, species that spend a considerable amount of time underground in burrows or that forage under loose soil and vegetation; (iv) *aquatic*, species that spend most or all of their time in an aquatic environment and often show anatomical specializations for swimming (e.g. sea snakes); and (v) *semi-aquatic*, species that spend considerable amounts of time in the water, but often emerge to feed, bask or reproduce (e.g. *Eunectes*, *Natrix*).

The R scripts and settings used for our analyses are available in electronic supplementary material, S5.

Institutional abbreviations: AMNH, American Museum of Natural History, New York, NY, USA; MCZ, Museum of Comparative Zoology, Cambridge, MA, USA; QM, Queensland Museum, Brisbane, QLD, Australia; SAMA, South Australian Museum, Adelaide, SA, Australia; USNM, National Museum of Natural History, Washington, DC, USA.

## 3. Results

As noted above, the digital endocast of the inner ear of *Yurlunggur* was sufficiently complete for quantitative morphometric analysis, while that of *Wonambi* was too incomplete, and will be discussed qualitatively in the Discussion.

**Figure 3.**
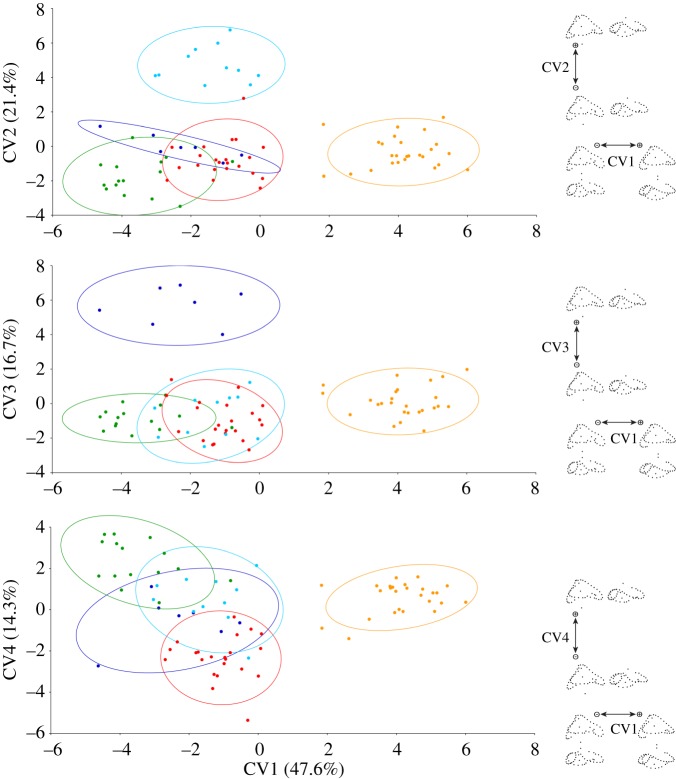
Distribution of the five ecological groups (81 taxa; all except *Yurlunggur*, whose ecology is unknown) in the CVs morphospace (ordinary CVA). Orange: fossorial/semifossorial taxa; cyan: semiaquatic taxa; blue: fully aquatic taxa; green: arboreal taxa; red: generalist taxa. 90% confidence ellipses for each ecological group are also shown. Procrustes landmark configurations towards the positive and negative extremes of each axis are shown on the right-hand side, in lateral and dorsal (to the right or below the former) views (anterior is to the right in all projections).

The results of the CVA (figure 3) show that ecological groups can be separated in shape space with a classification accuracy of 100% (*K* = 1). The percentage of variance explained by each canonical variate (CV) is: 47.6% for CV1, 20.5% for CV2, 17.7% for CV3, and 14.2% for CV4. The first two CVs separate semiaquatic (high values of CV2) and fossorial/semifossorial taxa (high values of CV1) from all other categories. In particular, while both semiaquatic and fossorial/semifossorial taxa have an enlarged saccular region, in semiaquatic forms the inner ear is characterized by a relatively larger lateral ampulla. High positive values of the third CV (CV3 > 3) distinguish (fully) aquatic taxa from the rest, and this translates morphologically in the combination of a relatively smaller saccular region, a shorter anterior semicircular canal, and a more mediolaterally compressed inner ear as a whole. Positive values of CV4 appear to be typical of arboreal forms, while negative values are typical of generalists. Morphologically, this corresponds to a relatively larger area enclosed by the anterior semicircular canal in arboreal forms, where the anterodorsal margin of the canal tends to be convex dorsally rather than concave.

The PCA of the inner ears from juveniles and adults of eleven different species showed that while some taxa show considerable ontogenetic shape change (e.g. *Varanus*), others show little such transformation (e.g. *Boiga*) (electronic supplementary material S2, figure S3). There is no common trajectory in the shape space defined by PC1 and PC2: the trajectories varied stochastically in length, axis orientation and direction (electronic supplementary material S2, figure S3). However, in the shape space defined by PC1 and PC3, several taxa had trajectories with similar orientation and direction, namely the lizards *Ctenophorus* and *Varanus* (both trending towards more positive values of PC3 and slightly more negative values of PC1), and the snakes *Acrochordus, Anilios, Aspidites, Cerberus, Cylindrophis* and *Notechis* (all trending towards more positive values of both PC1 and PC3). Interestingly, the snakes *Candoia* and *Boiga* have trajectories that go in opposite directions compared to all other snakes, which indicates lack of a consistent ontogenetic pattern across snakes as a whole.

Tests for phylogenetic signal found a statistically significant correlation between evolutionary history and shapes regardless of the phylogeny adopted (the null hypothesis of no phylogenetic signal present was rejected; Tree 1: *K* = 0.408, *p* = 0.0001; Trees 2 and 3: *K* = 0.410, *p* = 0.0001).

Both ordinary and phylogenetically informed Procrustes ANOVA found a statistically significant correlation between shapes and ecology (the null hypothesis of no difference between group means was rejected; ordinary Procrustes ANOVA: *F*_79_ = 3.4578, *p* = 0.0001, Rsq = 0.156; phylogenetic Procrustes ANOVA: *F*_79_ = 4.3988, *p* = 0.002, Rsq = 0.190). In other words, the variability between groups is significantly more than that expected based on variability within groups.

**Figure 4.**
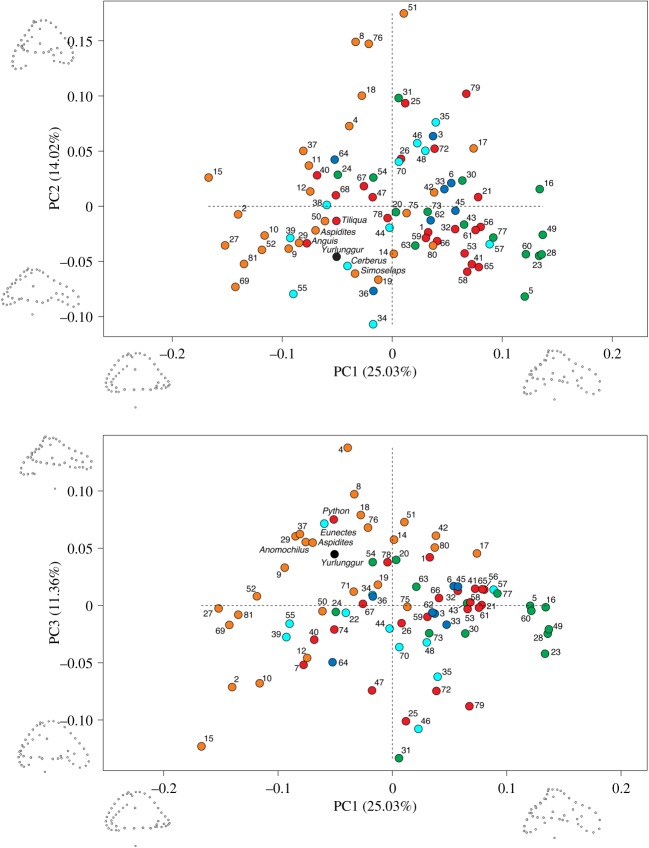
Distribution of the 82 inner ear endocasts in the morphospace defined by the first three principal components (ordinary PCA). Projections of the Procrustes landmark configurations corresponding to the positive and negative extremes of each axis are also shown (all projections are in lateral view, anterior to the right). Colour coding is the same as in figure 3, orange: fossorial/semifossorial taxa; cyan: semiaquatic taxa; blue: fully aquatic taxa; green: arboreal taxa; red: generalist taxa. The names of the taxa that are closest to *Yurlunggur* are shown; for all other taxa, see electronic supplementary material S4, table S2, where a species name is provided for each number.

The results of our ordinary PCA (figure 4) show that the first three components (PCs) explain approximately 50% of the variance. In the plot of PC1 versus PC2, *Yurlunggur* falls closest to a semiaquatic snake (the homalopsid *Cerberus*), while in the plot of PC1 versus PC3, *Yurlunggur* is surrounded mostly by fossorial/semifossorial taxa, but the semiaquatic *Eunectes* and the generalist *Python* are also quite proximal.

**Figure 5.**
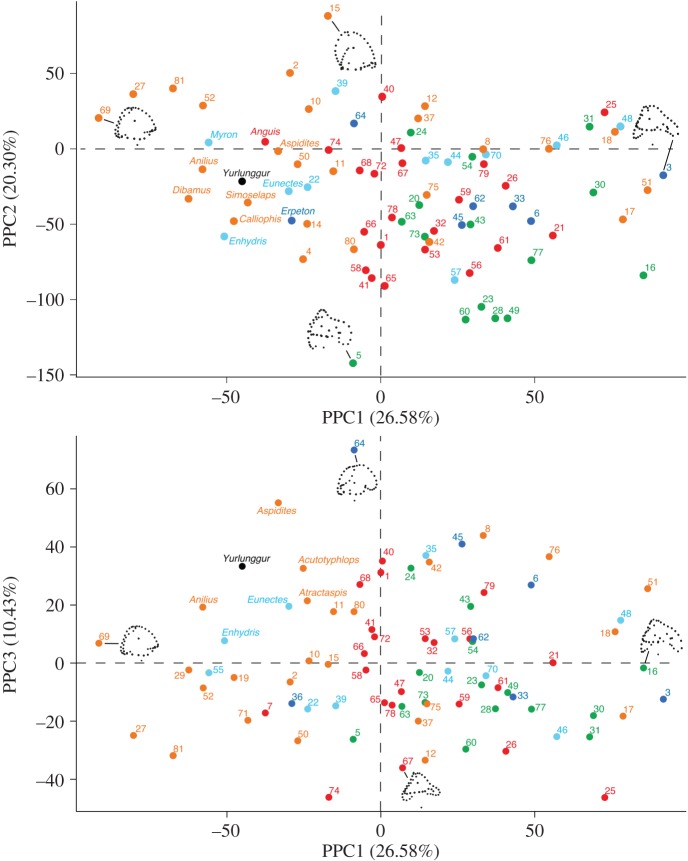
Distribution of the 82 inner ear endocasts in the morphospace defined by the first three phylogenetic principal components (PPCs from PPCA based on Tree 1). Projections of the Procrustes landmark configurations of actual specimens are shown towards the positive and negative extremes of each axis and as close as possible to the origin of the orthogonal axis (all projections are in lateral view, anterior to the right). Colour coding is the same as in figure 3, orange: fossorial/semifossorial taxa; cyan: semiaquatic taxa; blue: fully aquatic taxa; green: arboreal taxa; red: generalist taxa. The names of the taxa that are closest to *Yurlunggur* in each plot are shown; for all other taxa, see electronic supplementary material S4, table S2, where a species name is provided for each number.

In the PPCA (figure 5), the first three components explain approximately 57% of the variance. Both in the plot of PPC1 versus PPC2 and in that of PPC1 versus PPC3, *Yurlunggur* is surrounded mostly by semiaquatic and fossorial/semifossorial taxa. The plots of the PPCAs based on the three alternative tree topologies (Tree 1, Tree 2 and Tree 3) were very similar (result shown is from Tree 1).

No statistically significant correlation was found between size (measured as CS) and the PC1 of either the ordinary or the phylogenetic PCA (based on Tree 1; values of PC1 based on Trees 1, 2 and 3 were almost identical), regardless of the method adopted (for ordinary PC1: Pearson's method, *t*_80_ = −0.73174, corr = −0.08153896, *p* = 0.4665; Spearman's method, *S* = 81068, ρ = 0.1176848, *p*-value = 0.2917; Kendall's method, *z* = 1.1813, τ = 0.08882867, *p*-value = 0.2375; for phylogenetic PC1: Pearson's method, *t*_80_ = −1.0165, corr = −0.1129216, *p* = 0.3125; Spearman's method, *S* = 88254, ρ = 0.03947497, *p*-value = 0.7243; Kendall's method, *z* = 0.37241, τ = 0.02800361, *p*-value = 0.7096).

The classification tests using the typicality probability function and the scores of the first two PCs (table 1) indicate that *Yurlunggur* is closest to semiaquatic forms (highest probability of 67%, second highest being fossorial/semifossorial at 59.4%) when the scores are from the ordinary PCA, and is closest to semifossorial forms when the scores are from the phylogenetic PCA (based on Tree 1) (highest probability of 64.5%, second highest being semiaquatic at 42.8%); note that values do not add up to 100% because typicality probabilities are calculated for each group independently.

**Table 1.** Typicality probabilities of *Yurlunggur* based on the scores from the first two ordinary principal components (PCs), and based on the scores from the first two phylogenetic principal components (PPCs). Values are shown for when ‘aquatic’ and ‘semiaquatic’ are considered as separate categories (first two rows) and when they are merged into the same category (aquatic + semiaquatic) (bottom two rows).

	generalist (%)	arboreal (%)	fossorial/ semifossorial (%)	aquatic (%)	semiaquatic (%)
prob. based on PCs 1–2	38	14.1	59.4	33.9	*67*
prob. based on PPCs 1–2	32.7	9.2	*64.5*	17	42.8
prob. based on PCs 1–2	38.3	14.3	*59.1*	53.7	
prob. based on PPCs 1–2	33.2	9.6	*64.3*	31.9	

Owing to inconsistencies and lack of data in the ecological literature, it was often difficult to determine whether particular species were fully fossorial or semifossorial (as already discussed in [3], this would be possible only for a few of the best-documented cases), hence a single ‘fossorial’ category was used. However, we could readily separate fully aquatic and semiaquatic categories. We wanted to test whether merging fully aquatic and semiaquatic taxa into one category, thus better balancing out the number of taxa across all the ecological categories, would affect the classification of *Yurlunggur*. Our classification tests after doing so placed *Yurlunggur* in the fossorial category with the highest probability regardless of whether the scores were from the ordinary (59.1% probability) or the phylogenetic PCA (64.3% probability). However, a classification into the category ‘fully aquatic + semiaquatic’ was not far behind (53.7% for ordinary PCA and 31.9% for PPCA).

## 4. Discussion and conclusion

Prior to this study, it was becoming clear that interpreting causation for the variation in inner ear morphology in squamates is not a straightforward process. While ecology has a significant role in shaping inner ear morphology [1], phylogenetic constraint has also a strong influence [3]. Our results indicate that a third source of inner ear variation, ontogeny, may also be important. We observed ontogenetic trajectories of considerable length for some taxa (e.g. *Varanus*) relative to others (e.g. *Boiga*) (electronic supplementary material S2, figure S3). The length of some of these trajectories in the morphospace defined by PC1 and PC2, and also in that of PC1 and PC3, was greater than some of the distances separating different species. This implies that due care needs to be taken when applying morphometric methods to the inner ear of squamate reptiles in situations when the ontogenetic stage (i.e. juvenile versus adult) of a specimen is not clear. Luckily, the fossil specimen of *Yurlunggur* that we examined (QMF45111) is clearly an adult, based both on degree of ossification of its skull bones and overall size of the associated vertebrae, which fall in the upper range for the genus [9].

**Figure 6.**
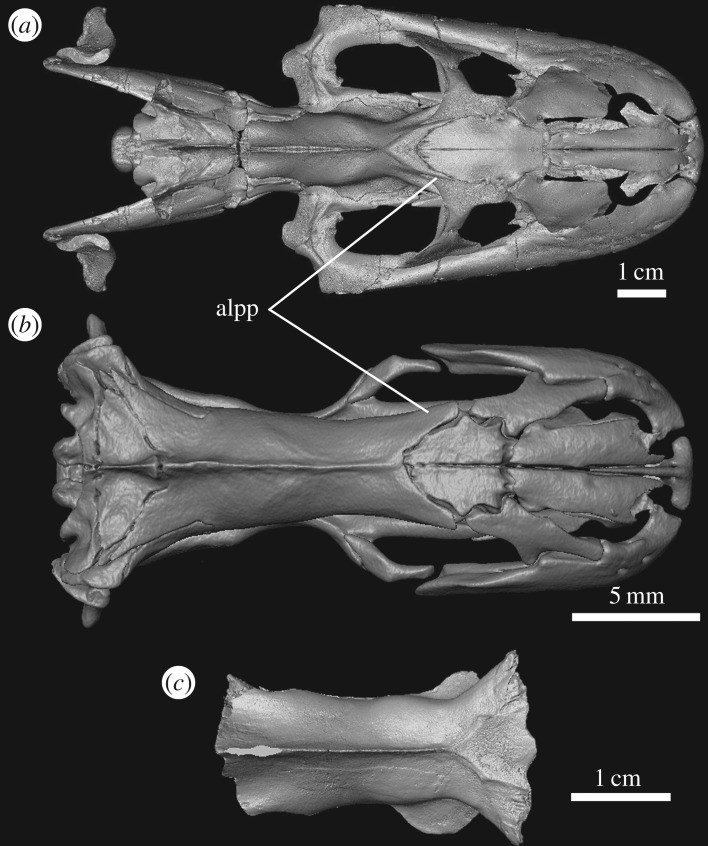
Comparison between (*a*) the skull of *Yurlunggur* sp. (reconstructed from QMF45 111–45 391) and (*b*) the skull of the burrowing snake *Anlius scytale* (USNM 204078) (both skulls are in dorsal view, anterior to the right). Note the bilateral anterolateral processes on the parietal (alpp) clasping the frontals in both snakes. In (*c*), the parietal of *Wonambi* (SAMA P27777) is also shown for comparison (dorsal view, anterior to the right).

The inner ear morphology of *Yurlunggur* resembles most closely that of fossorial/semifossorial taxa (e.g. *Simoselaps*, *Anilius*, *Aspidites*) as well as semiaquatic taxa (e.g. *Cerberus*, *Eunectes*). A semiaquatic ecology can be readily accepted for a large snake (estimated total length of approx. 5 m), but semifossorial habits may be harder to envision due to relatively large size. However, recent studies on the Australian python *Aspidites* (total length 2 m or more [51]) have shown that even fairly large snakes can actively burrow in search for prey [52]. Semifossorial habits in *Yurlunggur* are also supported by some cranial and postcranial features that are typically associated with fossorial behaviour in modern snakes. In particular, *Yurlunggur* has two anterolateral processes on the parietal that clasp the frontal and apparently reinforce the frontoparietal suture in a fashion very similar to what has evolved convergently in several fossorial and semifossorial snake lineages, for example *Anilius*, *Cylindrophis*, uropeltids, *Micrurus* and *Simoselaps* (figure 6). The overall skull morphology of *Yurlunggur* (figures 1 and 6) is, however, indicative more of an occasionally semifossorial lifestyle rather than of truly fossorial habits, because truly burrowing snakes (e.g. uropeltids, scolecophidians, *Anilius* and *Cylindrophis*) are typically characterized by small size, small eyes (*Yurlunggur* has relatively large orbits; figures 1 and 6), small gape, narrow head and a snout that is firmly connected to the rest of the skull [53]. However, some fossorial snakes lack these specializations and retain a relatively mobile (kinetic) skull (e.g. *Aspidites ramsayi* and *Aspidelaps scutatus* [52,53]).

**Figure 7.**
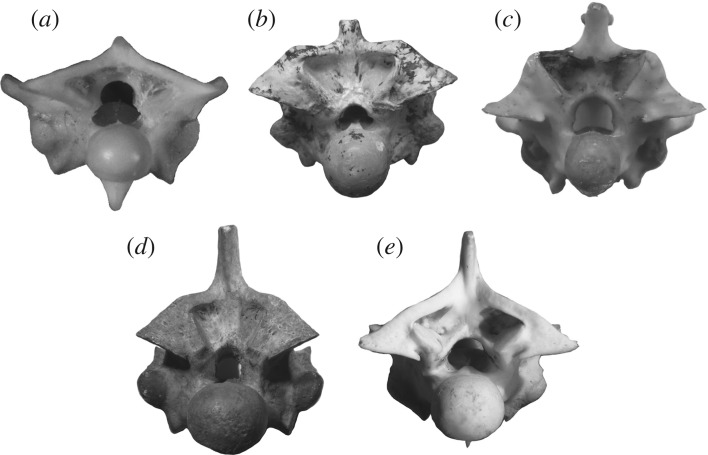
Posterior views of mid-dorsal vertebrae of (*a*) *Anilius scytale* (MCZ R19537), (*b*) *Yurlunggur* sp. (QM WH04), (*c*) *Aspidites ramsayi* (SAMA R68138), (*d*) *Wonambi naracoortensis* (SAMA P16168, type) and (*e*) *Eunectes murinus* (AMNH R29350). Note how the height of the neural spine of *Yurlunggur* is intermediate between those of the fossorial snake *Anilius* and the semifossorial *Aspidites*, while the neural spine of *Wonambi* is much taller and resembles that of the semiaquatic *Eunectes*. Images are not to scale.

Another feature that is indicative of semifossorial habits in *Yurlunggur* can be found in the postcranium, and specifically in the shape of the neural spines, which are typically very low in this genus, especially when compared with the neural spines of *Wonambi*. Low neural spines are again typical of fossorial or semifossorial habits in modern species [53] (figure 7). Due to the fact that the neural spines of the closely related *Wonambi* look quite different, a phylogenetic constraint can easily be ruled out. In particular, the neural spines on the mid-trunk vertebrae of *Yurlunggur* look intermediate in size between those of *Anilius*, a fossorial species, and those of the semifossorial *Aspidites*. Interestingly *Anilius*, besides being fossorial, also has semiaquatic habits, preying largely on freshwater eels [54]. This would be consistent with our findings suggesting a mixed ecology for *Yurlunggur*.

Therefore, features of the inner ear, skull and vertebrae suggest that *Yurlunggur* was likely adapted to a mixed semiaquatic and semifossorial lifestyle; its ecology may have been similar to that of modern red pipe snakes (*Anilius scytale*), which burrow but also hunt for prey in rivers [54]. Because of its skull structure and large size, burrowing behaviour in *Yurlunggur* was likely limited to occasional digging in loose or soft soil, like that of woma pythons *Aspidites ramsayi* [52].

The incomplete nature of the inner ear endocast of *Wonambi*, which is missing the whole upper portion, precluded inclusion in the quantitative geometric morphometric study. However, a general comparison based on gross morphology is still possible: most importantly, some of the main features of the inner ear, skull and postcranium of *Wonambi* can still be compared with homologous structures in *Yurlunggur*.

Compared with the inner ear of *Yurlunggur*, that of *Wonambi* (figure 1) has a relatively smaller saccular portion, a much shorter lateral semicircular canal, and much taller anterior and posterior semicircular canals based on the preserved portions. Given that *Wonambi* and *Yurlunggur* are closely related Australian madtsoiids, these differences likely reflect adaptation; a similar situation has been documented in closely related *Anolis* [2]. Interestingly, on the skull roof, the parietal of *Wonambi* lacks the anterolateral processes visible in *Yurlunggur* and typical of fossorial and semifossorial snakes (see above). Moreover, the neural spines of mid-trunk vertebrae of *Wonambi* are relatively much taller than those of *Yurlunggur* and semifossorial taxa; their relative height is similar to that observed in the semiaquatic anaconda *Eunectes* (figure 7), and is consistent with semiaquatic habits, although this can only remain speculative in the absence of a comprehensive survey of the relative heights and morphology of the neural spines of snakes (while low neural spines are generally accepted as an indicator of fossorial/semifossorial habits [53], a tall neural spine may be associated with multiple habitats, e.g. [55]).

There is support for the inference that these two snakes had distinct environmental preferences if we compare the relevant palaeoclimatic information available for the Late Oligocene and Early Miocene at Riversleigh in northern Queensland, the locality of *Yurlunggu*r [8], and that available for the Late Pleistocene in southern Australia, where *W. naracoortensis* has been found [7]. While *Yurlunggur* likely lived in a warm mesic forest habitat (e.g. [56]), *W. naracoortensis* occupied much cooler and drier regions of the Australian continent (e.g. [57]). A geologically rapid shift towards drier and cooler conditions in the mid-Miocene [57] may have been responsible for the disappearance of *Yurlunggur* and similar taxa at Riversleigh, especially if they were semiaquatic, while the ability of *Wonambi* to live in much cooler and drier habitats may explain its much longer and widespread persistence in the fossil record despite the increasing aridification of the Australian continent.

Finally, the diversity of inner ear, skull and postcranial morphology evident in *Yurlunggur* and *Wonambi* suggests considerable ecological diversity and plasticity across madtsoiids and other extinct basal snake lineages. Such disparity should not be surprising, given the known history of madtsoiids spans approximately 100 Myr, which is roughly equivalent to the inferred age of modern (crown) snakes. This means caution is warranted when using single fossil snakes to make broad extrapolations about early snake biology.

## Supplementary Material

Supplementary material S1 - Table S1

## Supplementary Material

Supplementary material S2

## Supplementary Material

Supplementary material S3

## Supplementary Material

Supplementary material S4 - Table S2

## Supplementary Material

Supplementary material S5

## Data accessibility

Additional resources are available as electronic supplementary material from the Dryad Digital Repository at http://dx.doi.org/10.5061/dryad.5gs6p [58].
