# Supplementary material for: Palaeoecological inferences for the fossil Australian snakes Yurlunggur and Wonambi (Serpentes, Madtsoiidae)
Source: R Soc Open Sci. 2018 Mar 14;5(3):172012. doi: 10.1098/rsos.172012 (PMC5882723; doi:10.1098/rsos.172012)
Supplement: Supplementary material S2 [file rsos172012supp2.pdf]

Royal Society Open Science

Palaeoecological inferences for the fossil Australian snakes *Yurlunggur* and *Wonambi* (Serpentes, Madtsoiidae)

Alessandro Palci[1,2], Mark N. Hutchinson[1,2,3], Michael W. Caldwell[4], John Scanlon[5], and Michael S. Y. Lee[1,2]

1, South Australian Museum, Adelaide, South Australia, Australia

2, College of Science and Engineering, Flinders University, Adelaide, South Australia, Australia

3, School of Biological Sciences, University of Adelaide, Adelaide, South Australia, Australia

4, Department of Biological Sciences, University of Alberta, Edmonton, Alberta, Canada

5, School of Biological, Earth and Environmental Sciences, University of New South Wales, Kensington, New South Wales, Australia

Supplementary Material S2: Landmarking Procedure, Replacement Taxa, and Supplementary Figures

Procedure followed in the placing of landmarks and semilandmarks (after Palci et al., 2017):

The first landmark (landmark 1) was fixed and placed at the notch marked by the point where a branch of the auditory nerve (anterior branch of the VIII nerve) [7] meets the utriculus (Fig. S1A). This was followed by a series of sliding semilandmarks, spaced as evenly as possible, along the anterior semicircular canal, on the line of maximum curvature facing away from the sacculus (Fig. S1A–B). The landmark located on the top of the crus communis was a fixed landmark (landmark 16). A second series of evenly spaced sliding semilandmarks was placed along the posterior semicircular canal, again along the line of maximum curvature facing away from the sacculus (Fig. S1B,C). This series terminated in a fixed landmark (landmark 30) located at a point where the posterior ampulla meets the sacculus, typically dorsal to the start of the perilymphatic duct (the canal that leads to the fenestra rotunda), and

at a point where the ampulla is considerably tapered posteriorly. The next landmark (landmark 31) was also fixed and located on the dorsal end of the groove that separates lateral and anterior ampulla, with the lateral semicircular canal placed horizontally (Fig. S1D). This was followed by a series of evenly spaced sliding semilandmarks along the lateral semicircular canal in a posterior direction and located dorsolaterally along the canal. This series terminated in a fixed landmark (landmark 45) located at the posterior end of the lateral semicircular canal, which is typically marked by a more or less pronounced concavity at the base of the crus communis (Fig. S1E). The next two landmarks were fixed, and placed after the digital endocast of the inner ear was positioned following two simple steps: (i) the semicircular canals were aligned tangent to an imaginary vertical plane located to the left of the endocast (Fig. S1F) and (ii) the endocast was rotated about 90° counterclockwise (as seen from a dorsal perspective), so that the lateral surface of the sacculus was facing the observer (Fig. S1G,H). This step ensured that the sacculus was observed from a consistent angle when placing the next two landmarks. Landmark 46 was placed at the ventral end of the groove that separates anterior and posterior semicircular canals at the level of the crus communis; while landmark 47 was placed approximately in the centre of the sacculus, which seen in projection from such angle can usually be approximated to a circle.

When this was not the case (i.e. the sacculus was far from hemispherical) the landmark was placed in the circumcentre, i.e. in a point that would correspond to the centre of the circumscribed circle that best fits the outline of the sacculus in the specific view obtained following steps 1 and 2. The last fixed landmark (landmark 48) was placed at the bottom of the lagena, typically at the point of maximum curvature, but excluding sutures, since the latter can create an artificial relief (Fig. S1I).

It should be noted that in some species the posterior semicircular canal partially intersects the lateral semicircular canal. This can create problems in the placing of evenly spaced semilandmarks along the lateral semicircular canal (landmarks 32–44). We avoided placing a semilandmark medial to the intersection (i.e. medial to the posterior semicircular canal), and placed one semilandmark anterior to the intersection and another posterior to it. This was considered to be the best solution, because with the adoption of sliding semilandmarks the spacing does not have to be strictly even, as long as the general curvature defined by these points remains representative of the general curvature of the shape that they are meant to represent.

The semilandmarks were approximately evenly spaced between pairs of fixed landmarks, but the actual spacing varied between species (e.g. some species have a much longer anterior semicircular canal relative to others, which nonetheless needs to accommodate the same number of semilandmarks).

Replacement taxa:

The phylogenetic tree adopted for the various phylogenetic tests (phylogenetic signal, phylogenetic ANOVA, and phylogenetic PCA) using extant taxa was obtained from Zheng and Wiens (2016). Whenever one of our selected species was missing in the tree, we selected a close relative:

Ahaetulla nasuta was selected instead of *A. prasina*;
Brachyurophis semifasciatus instead of *B. australis*;
Calliophis melanurus instead of *C. maculiceps*;
Dendrelaphis bifrenalis instead of *D. calligastra*;
Dipsas albifrons instead of *D. pavonina*;
Draco dussumieri instead of *D. walkeri*;
Lerista aericeps instead of *L. timida*;
Liotyphlops albirostris instead of *L. beui*;
Pseudonaja modesta instead of *P. mengdeni*;
Pygopus lepidopus instead of *P. schraderi*;
Rhinophis blithyi instead of *R. saffragamus*;
Trilepida macrolepis instead of *T. dimidiatum*;
Vermicella calonotus instead of *V. annulata*).

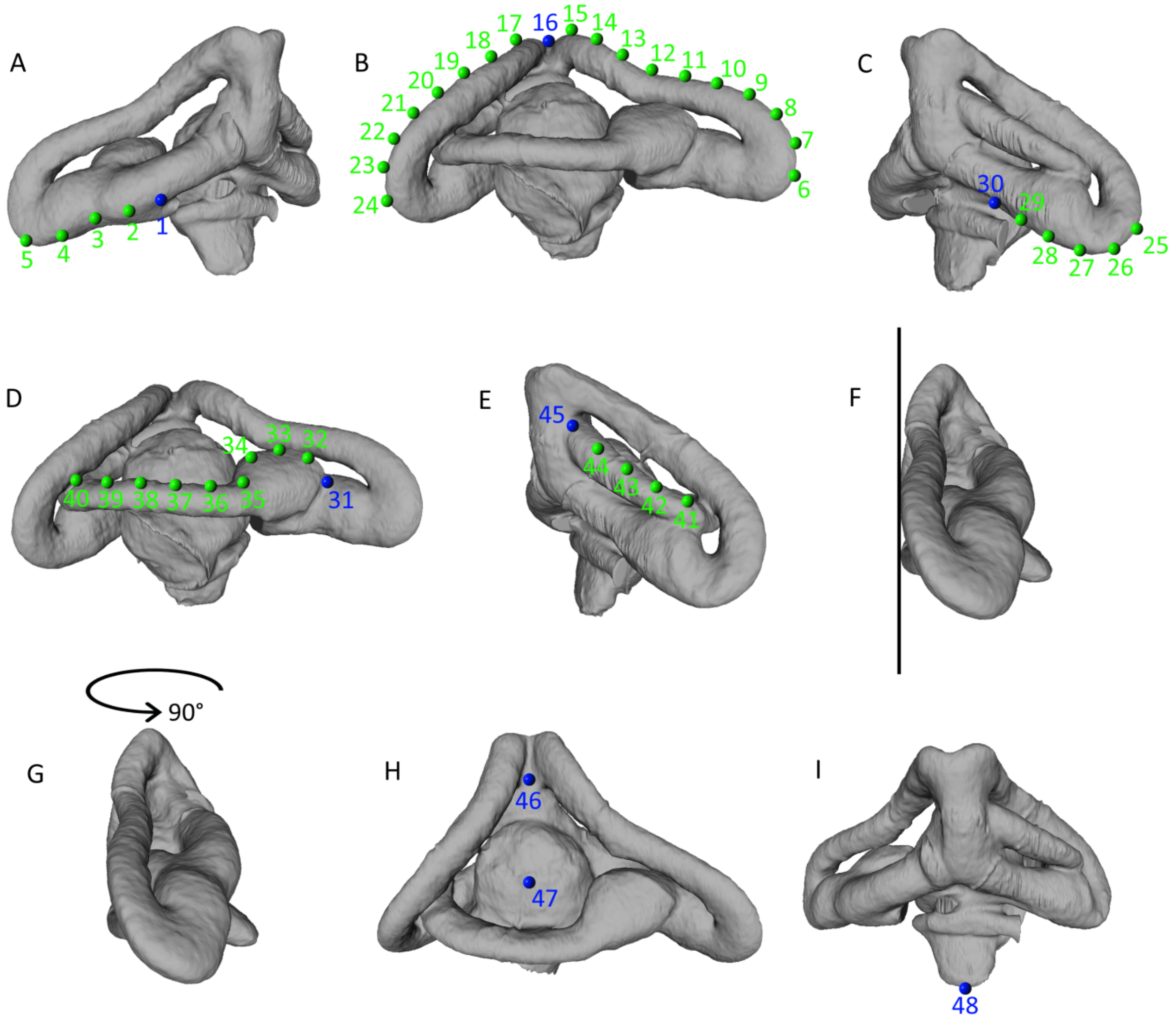

FIGURE S1: Position of landmarks (blue) and semilandmarks (green) on the inner ear endocast of *Lycodon aulicus* (SAMA R36823).

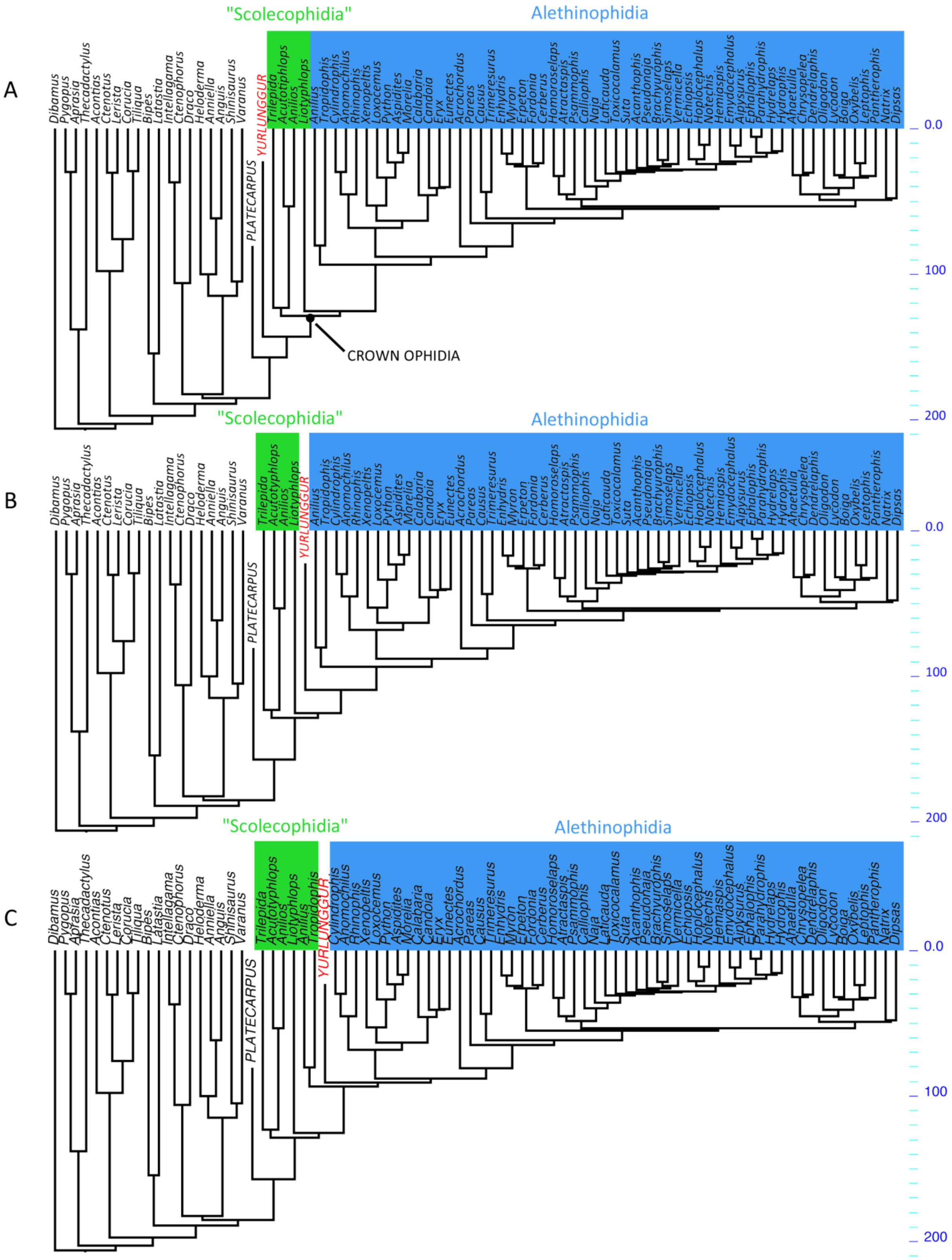

A
"Scolecophidia"
Alethinophidia
Dibamus Pygopus Aprasia Thecadactylus Acontias Ctenotus Lerista Corucia Tiliqua Bipes Latastia Intellagama Ctenophorus Draco Heloderma Anniella Anguis Shinisaurus Varanus
PLATECARPUS
YURLUNGGUR
Trilepida Acutotyphlops Anilios Liotyphlops
Anilius Tropidophis Cylindrophis Anomochilus Rhinophis Xenopeltis Loxocemus Python Aspidites Morelia Calabaria Candoia Eryx Eunectes Acrochordus Pareas Causus Trimeresurus Enhydris Myron Erpeton Fordonia Cerberus Homoroselaps Atractaspis Psammophis Calliophis Naja Laticauda Toxicocalamus Suta Acanthophis Pseudonaja Brachyurophis Simoselaps Vermicella Echiopsis Hoplocephalus Notechis Hemiaspis Emydocephalus Aipysurus Ephalophis Parahydrophis Hydrelaps Hydrophis Ahaetulla Chrysopelea Dendrelaphis Oligodon Lycodon Boiga Oxybelis Leptophis Pantherophis Natrix Dipsas
CROWN OPHIDIA
0.0
100
200
B
"Scolecophidia"
Alethinophidia
Dibamus Pygopus Aprasia Thecadactylus Acontias Ctenotus Lerista Corucia Tiliqua Bipes Latastia Intellagama Ctenophorus Draco Heloderma Anniella Anguis Shinisaurus Varanus
PLATECARPUS
Trilepida Acutotyphlops Anilios Liotyphlops
YURLUNGGUR
Anilius Tropidophis Cylindrophis Anomochilus Rhinophis Xenopeltis Loxocemus Python Aspidites Morelia Calabaria Candoia Eryx Eunectes Acrochordus Pareas Causus Trimeresurus Enhydris Myron Erpeton Fordonia Cerberus Homoroselaps Atractaspis Psammophis Calliophis Naja Laticauda Toxicocalamus Suta Acanthophis Pseudonaja Brachyurophis Simoselaps Vermicella Echiopsis Hoplocephalus Notechis Hemiaspis Emydocephalus Aipysurus Ephalophis Parahydrophis Hydrelaps Hydrophis Ahaetulla Chrysopelea Dendrelaphis Oligodon Lycodon Boiga Oxybelis Leptophis Pantherophis Natrix Dipsas
0.0
100
200
C
"Scolecophidia"
Alethinophidia
Dibamus Pygopus Aprasia Thecadactylus Acontias Ctenotus Lerista Corucia Tiliqua Bipes Latastia Intellagama Ctenophorus Draco Heloderma Anniella Anguis Shinisaurus Varanus
PLATECARPUS
Trilepida Acutotyphlops Anilios Liotyphlops Anilius Tropidophis
YURLUNGGUR
Cylindrophis Anomochilus Rhinophis Xenopeltis Loxocemus Python Aspidites Morelia Calabaria Candoia Eryx Eunectes Acrochordus Pareas Causus Trimeresurus Enhydris Myron Erpeton Fordonia Cerberus Homoroselaps Atractaspis Psammophis Calliophis Naja Laticauda Toxicocalamus Suta Acanthophis Pseudonaja Brachyurophis Simoselaps Vermicella Echiopsis Hoplocephalus Notechis Hemiaspis Emydocephalus Aipysurus Ephalophis Parahydrophis Hydrelaps Hydrophis Ahaetulla Chrysopelea Dendrelaphis Oligodon Lycodon Boiga Oxybelis Leptophis Pantherophis Natrix Dipsas
0.0
100
200

FIGURE S2. Trees adopted for the tests of phylogenetic signal. A) Tree1, *Yurlunggur* is positioned as a stem ophidian; B) *Yurlunggur* is positioned as a stem alethinophidian; C) *Yurlunggur* is placed within Alethinophidia. Branch lengths are proportional to time. Tree topology of extant taxa from Zheng and Wiens (2016). See main text for details about the placement of the fossil taxa *Platecarpus* and *Yurlunggur*.

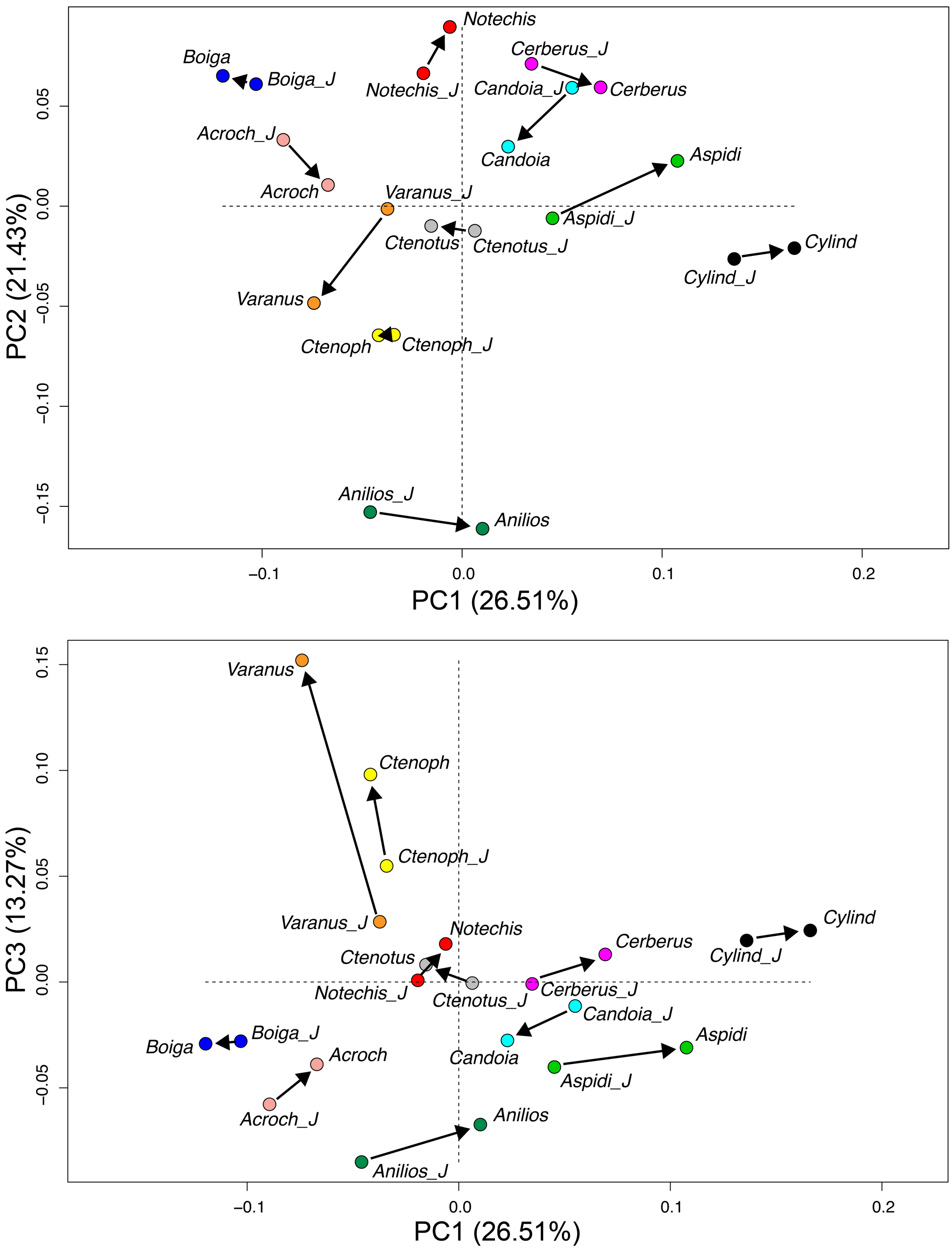

Notechis
Cerberus_J
Boiga
Boiga_J
Notechis_J
Candoia_J
Cerberus
Acroch_J
Candoia
Aspidi
Acroch
Varanus_J
Aspidi_J
Ctenotus
Ctenotus_J
Cylind
Cylind_J
Varanus
Ctenoph
Ctenoph_J
Anilios_J
Anilios
0.05
0.00
−0.05
−0.10
−0.15
PC2 (21.43%)
−0.1
0.0
0.1
0.2
PC1 (26.51%)
Varanus
Ctenoph
Ctenoph_J
Varanus_J
Notechis
Cerberus
Cylind
Cylind_J
Ctenotus
Notechis_J
Ctenotus_J
Cerberus_J
Candoia_J
Boiga_J
Boiga
Acroch
Candoia
Aspidi
Aspidi_J
Acroch_J
Anilios
Anilios_J
0.15
0.10
0.05
0.00
−0.05
PC3 (13.27%)
−0.1
0.0
0.1
0.2
PC1 (26.51%)

FIGURE S3. Distribution of juvenile-adult pairs in the morphospace of the first three principal components (ordinary PCA). Note the lack of a common ontogenetic pattern in the plot of PC1 vs PC2, but the presence of some similar ontogenetic trajectories in the plot of PC1 vs PC3. However, lack of consistent trajectories both within lizards and within snakes indicates that there is no common ontogenetic pattern shared by all taxa, even within the same group (i.e., lizards or snakes). Snakes: *Acroch*, *Acrochordus arafurae; Anilios*, *Anilios bicolor*; *Aspidi*, *Aspidites ramsayi; Boiga*, *Boiga irregularis; Candoia*, *Candoia carinata; Cerberus, Cerberus rhynchops; Cylind*, *Cylindrophis ruffus; J, juvenile; Notech, Notechis scutatus.* Lizards: *Ctenotus*, *Ctenotus spaldingi; Ctenoph*, *Ctenophorus decresii; Varanus*, *Varanus gilleni.*

References:

Palci A, Hutchinson MN, Caldwell MW, Lee MSY. 2017. The morphology of the inner ear of squamate reptiles and its bearing on the origin of snakes. *R. Soc. Open Sci.* **4**, 170685. (doi:10.1098/rsos.170685)

Zheng Y,Wiens JJ. 2016 Combining phylogenomic and supermatrix approaches, and a time-calibrated phylogeny for squamate reptiles (lizards and snakes) based on 52 genes and 4,162 species. *Mol. Phylogenet. Evol.* **94**, 537–547. (doi:10.1016/j.ympev. 2015.10.009)
